# Six-Year Incidence and Risk Factors of Age-Related Macular Degeneration in Singaporean Indians: The Singapore Indian Eye Study

**DOI:** 10.1038/s41598-018-27202-w

**Published:** 2018-06-11

**Authors:** Valencia Hui Xian Foo, Yasuo Yanagi, Quang Duc Nguyen, Charumathi Sabanayagam, Sing Hui Lim, Kumari Neelam, Jie Jin Wang, Paul Mitchell, Ching-Yu Cheng, Tien Yin Wong, Chui Ming Gemmy Cheung

**Affiliations:** 1 Singapore Eye Research Institute, Singapore National Eye Centre, Singapore, Singapore; 2 Ophthalmology & Visual Sciences Academic Clinical Program (Eye ACP), Duke-NUS Medical School, Singapore, Singapore; 3 Centre for Quantitative Medicine, Duke-NUS Medical School, Singapore, Singapore; 4 Singapore National Eye Centre, Singapore, Singapore; 5 Academic Medicine Research Institute, Duke-NUS Medical School, Singapore, Singapore; 6 Centre for Vision Research, Westmead Millennium Institute of Medical Research, University of Sydney, Westmead, Sydney Australia

## Abstract

We aimed to determine the 6-year incidence and risk factors of age-related macular degeneration (AMD) in first and second generations of Singaporean Indians. Baseline examination was conducted in 2007–9 and 6-year propsective follow-up examination of this Indian population in 2013–5. All participants underwent interviews with questionnaires and comprehensive medical and eye examinations. Incidence was age-standardized to Singaporean 2010 census. Risk factors associated with AMD incidence were assessed and compared between first and second generations of immigrants. Among 2200 persons who participated in the follow-up examination (75.5% response rate), gradable fundus photographs were available in 2105. The 6-year age-standardized incidences of early and late AMD were 5.26% and 0.51% respectively. Incident early AMD was associated with cardiovascular disease history (HR 1.59, 95% CI 1.04–2.45), underweight body mass index (BMI) (HR 3.12, 95% CI 1.37–7.14) (BMI of <18.5 vs 18.51–25 kg/m2), heavy alcohol drinking (HR 3.14 95% CI 1.25–7.89) and ARMS2 rs3750847 homozygous genetic loci carrier (HR 2.52, 95% CI 1.59–3.99). We found a relatively low incidence of early AMD in this Singaporean Indian population compared to Caucasian populations. Both first and second-generation Indian immigrants have similar incidence and risk factor patterns for early AMD.

## Introduction

Age-related macular degeneration (AMD) is the leading cause of severe visual impairment globally, accounting for 8.7% of all blindness worldwide^1–6^. It is crucial to understand the population-specific incidence and risk factors of AMD in planning strategies for future healthcare provision of ageing populations. Earlier studies have reported the incidence of early and late AMD ranging from 5 to 10-year follow-ups in the Caucasian^6–12^ Chinese^13^, Japanese^14^ and Malay^15^ populations. A range of systemic and ocular risk factors for AMD have been reported, including cigarette smoking^16–22^, dyslipidemia^16,17,23,24^, and the presence of soft drusen and/or pigmentary abnormalities^25–36^. To the best of our knowledge, no previous prospective studies have been conducted in Indians to assess AMD incidence and its associated risk factors.

Immigrant populations from developing to developed countries may be influenced by changing lifestyles and environmental factors (e.g., Western type diets, increased incidence of smoking etc)^37,38^. For example, second-generation immigrants of Indians in Singapore^39^ and Australia^40^ have a higher prevalence of diabetes-related complications compared to first-generation immigrants likely due to the metabolic impact of a westernized diet^41,42^. With an increasing number of Asian Indians who have migrated across the world, studying the impact of immigration on AMD incidence may shed insight on further risk factors for AMD.

In addition, genes have been estimated to explain about 50% of the heritability of AMD. There are over 36 genetic loci discovered each with various roles in the development of AMD. In Asians, the genetic loci most strongly attributed to AMD development are the *Complement Factor H* (*CFH)* gene and *Age-related Macular Susceptibility 2 (ARMS2) / High-temperature requirement A-1 (HTRA1)* loci as reported in the Genetics in AMD in Asians (GAMA) consortium^43^. Earlier studies have shown that the *ARMS2* gene has a stronger influence on AMD development compared to the *CFH* gene^44,45^. Further work is needed to support this genetic association in the Indian population.

In view of these unanswered questions, we aimed to determine the 6-year incidence and ocular, systemic and genetic risk factors of early and late AMD in Singaporean Indians, and examined these differences between first and second-generation Indian immigrants.

## Methods

### Study Population

Our study utilised data from the Singapore Indian Eye Study (SINDI), a population-based cohort study of eligible Indian adults with baseline examination conducted from 2007 to 2009^16^. The recruitment methodology of SINDI has been described in detail elsewhere^16^. SINDI-2^46^ is the 6-year follow-up study (from 2013 to 2015) among Indian adults who participated in the baseline SINDI study. All 3400 participants from SINDI were sent invitation to attend the 6-year follow-up examinations at the Singapore Eye Research Institute (SERI) via telephone, by mail and/or by home visit.

The protocol used in SINDI-2 was identical to that of the SINDI baseline study. Of the 3400 participants from baseline, 486 participants were found to be ineligible to participate in SINDI-2 examination, with 201 deceased, 164 with terminal illnesses, 62 who have migrated, 40 who remained uncontactable, 13 with psychiatric illnesses and 6 who are prisoners. Of the remaining 2914 participants who were considered eligible for the complete follow-up examination, 2200 (75.5% response rate) participated in SINDI-2. After excluding 95 participants (4.3%) with ungradeable photos, data for a remaining 2,105 participants were included in this report (refer to Supplementary Table 1 for comparison between included and excluded patients).

Both baseline and follow-up study were approved by the Institutional Review Board of SingHealth (IRB Approval number: R933/42/2012), Singapore, and conducted in accordance with the Declaration of Helsinki. All participants provided written informed consent.

### Immigration Status

Participants were defined as ‘first-generation Indian immigrants’ if they were born in India and as ‘second-generation Indian immigrants’ if they were born in Singapore irrespective of the country of birth of their parents^47^. We have 793 first-generation immigrants, with 465 of them with parents from India, 290 of them with parents from Malaysia, 39 from other countries and 1 from Indonesia.

### Ophthalmic examination and AMD grading

A comprehensive eye examination was performed to obtain participants’ subjective refraction, distance best corrected visual acuity (BCVA) and near vision acuity. Auto-refraction, keratometry, ocular biometry, slit-lamp examination of the anterior segment and tonometry were carried out. Dilated fundus photographs of Early Treatment for Diabetic Retinopathy Study standard fundus fields 1 (centered on the optic disc) and 2 (centered on the fovea) were obtained for both eyes using a digital retinal camera (Canon CR-1 Mark-II Nonmydriatic Digital Retinal Camera, Canon).

Experienced graders in the Centre for Vision Research, University of Sydney, performed AMD grading using the Wisconsin Age-Related Maculopathy Grading System^48^. All photographs were graded initially in a masked manner, followed by a side-by-side grading of the baseline and six-year photographs. Early AMD as presence of either soft indistinct or reticular drusen or both soft, distinct drusen plus retinal pigment epithelium abnormalities. Late AMD was defined as the presence of neovascular AMD or geographic atrophy (GA). Neovascular AMD included serous or hemorrhagic detachment of the RPE or sensory retina, and the presence of subretinal or sub-RPE hemorrhages or subretinal fibrous scar tissue. GA was characterized by sharply edged, roughly round or oval areas of RPE hypopigmentation, with clearly visible choroidal vessels. The minimum area of GA was 175um in diameter or larger^48^.

### Systemic risk factor assessment

A detailed questionnaire-based interview was administered by trained interviewers with the information collected: contact and demographic information, education, income level, occupation, medical history and lifestyle factors (smoking, alcohol). Alcohol intake frequency was defined as non-drinker (0 days per week of drinking), moderate drinker (1–3 days a week of drinking) and heavy drinker (4–7 days a week of drinking). We did not have complete information on the units or the type of alcohol consumed per day in our population and hence this information was not included in our analysis. Participants’ heights were measured in centimeters using a wall-mounted measuring tape. Weight was measured in kilograms using a digital scale (SECA, model 7822321009: Vogel & Halke, Hamburg, Germany). Systolic and diastolic blood pressure were measured with a digital automatic blood pressure monitor (Dinamap model Pro Series DP110X-RW, 100V2; GE Medical Systems Information Technologies Inc., Milwaukee, USA) with the participant seated after 5 minutes of rest. Non-fasting venous blood was collected for Hemoglobin A1c (HbA1c), serum glucose, C-reactive protein (CRP) and lipid levels. All serum biochemistry tests were performed in the Singapore General Hospital Laboratory on the same day.

Hypertension was defined as systolic blood pressure ≥140 mmHg, diastolic blood pressure ≥90 mmHg or by physician diagnosis. Diabetes was defined as HbA1c >6.5%, casual glucose ≥11.1 mmol/L or use of diabetic medication. Chronic kidney disease was defined as an estimated glomerular filtration rate of less than 60 ml/minute/1.73 m, measured from serum creatinine^49^. Cardiovascular disease history includes a history of both coronary artery disease (acute myocardial infarction and angina) and stroke.

### Genotype

Genotyping was performed using Illumina Human OmniExpres or Human Hap610-Quad Beadchip. The commonly associated SNPs in the *CFH* and *ARMS2/HTRA-1* genes, namely rs1061170 and rs3750847, were not included in the Human610-Quad BeadChips. Hence, we proceeded to test other SNPs within the genes of interest i.e. *CFH* and *ARMS2/HTRA-1* for associations with AMD. We chose the SNP rs10801555 for CFH and rs3750847 for ARMS2 after confirming that they were in perfect linkage disequilibrium (LD; r^2^ = 1.0) with the Y402H variant (rs1061170) for *CFH* and rs3750847 for *ARMS2*^49^. Genetic data of these 2 SNPs was available in 1570 participants.

### Statistical Analysis

Baseline characteristics were compared using age-adjusted t-test between individuals who were followed-up and those who did not return for SINDI-2 examination or had ungradable retinal photographs.

Incidence estimates were standardized to the 2010 Singapore census population. Incident early AMD was defined by the appearance at follow-up of either indistinct soft or reticular drusen or the co-presence of both distinct soft drusen and retinal pigmentary abnormalities in either eye of persons in whom no early or late AMD was present at baseline. Incident late AMD was defined by the appearance at follow-up of neovascular AMD or GA in either eye of persons in whom no late AMD lesion was present at baseline.

The associations of potential risk factors with the incidence of early AMD were analysed in separate Generalized Estimating Equation (GEE) models that were adjusted for (1) age and gender (Model 1), and (2) smoking status, hypertension, serum CRP and additional variables that were statistically significant (P < 0.05) in Model 1. Risk factors for late AMD were not assessed due to a low number of cases. For first and second-generation multivariate analysis, multivariable-adjusted analysis including variables that were statistically significant (P < 0.05) in the univariate analysis was done with early AMD as the outcome.

We regarded P values of <0.05 from 2-sided tests to indicate statistical significance. All statistical analyses were performed using the Stata Statistical computer package (STATA Statistical Software, Version 12, Statacorp, College Station, Texas, USA) and R (R Core Team, R Foundation for Statistical Computing, Vienna, Austria).

### Data Availability

All data generated or analysed during this study are included in this published article.

### Synopsis

Cardiovascular disease, underweight BMI, alcohol drinking and ARMS2 rs3750847 homozygous carrier are risk factors for early age-related macular degeneration (AMD) in Indians. First and second-generation immigrants have similar incidence patterns and risk factors for early AMD.

## Results

We included 2105 participants with AMD grading for both baseline and six-year examinations. The baseline characteristics of participants are summarized in Table 1. Participants lost to follow-up at SINDI-2 were more likely to have slightly higher systolic and diastolic blood pressure, higher HbA1c and have lower socioeconomic status, defined as participants with primary or lower education only and individual monthly income <$2,000 Singapore dollars, at baseline, compared to those examined at SINDI-2. (p < 0.05 for all) (Supplementary Table 1).

**Table 1:** Baseline Characteristics of Participants and according to first and second generation.

Variable*	Participants at both examinations		First Generation		Second Generation		P value
	(n = 2105)		(n = 793)^†^		(n = 1312)^††^		
	Mean or n	SD or %	Mean or n (%)	SD or %	Mean or n (%)	SD or %	
Age (years)	56.20	9.07	59.54	10.22	54.18	7.62	<0.001
Sex, Male	1047	49.74	400	50.44	647	49.31	0.93
Hypertension, yes	1177	55.99	486	61.29	691	52.79	0.34
Systolic Blood Pressure, (mmHg)	134.11	19.30	135.97	19.56	132.99	19.06	0.29
Diastolic Blood Pressure, (mmHg)	77.75	10.11	77.11	9.77	78.14	10.30	0.38
Diabetes	570	27.17	235	29.67	335	25.65	0.18
Hba1c, %	6.36	1.32	6.33	1.22	6.38	1.37	0.04
Chronic Kidney Disease	105	5.16	49	6.36	56	4.43	0.08
Blood Creatinine (mmol/l)	0.85	0.25	0.86	0.24	0.85	0.26	0.32
History of Myocardial Infarction	162	7.70	72	9.09	90	6.86	0.13
History of Stroke	43	2.04	19	2.40	24	1.83	0.87
Body Mass Index (kg/m^2^)	26.20	4.48	25.82	3.98	26.44	4.74	0.01
Total Cholesterol (mg/dl)	5.21	1.07	5.07	1.04	5.29	1.08	<0.001
HDL Cholesterol (mg/dl)	1.06	0.31	1.05	0.31	1.07	0.30	0.01
LDL Cholesterol (mg/dl)	3.35	0.93	3.25	0.92	3.41	0.92	0.10
Triglycerides (mg/dl)	1.96	1.17	1.94	1.15	1.98	1.19	0.60
C-reactive Protein (mg/l), mgL	4.08	5.75	3.65	5.61	4.34	5.81	0.02
Formal Education, yes	1787	84.97	621	78.41	1166	88.94	0.58
Low income, <$2000	1440	70.24	545	70.96	895	69.81	0.28
Low Economic Status*, yes	967	47.19	366	47.72	601	46.88	0.02
Cigarette Smoking status							0.13
Never smoked	1570	74.58	624	78.69	946	72.10	
Current smoker	285	13.54	71	8.95	214	16.31	
Past Smoker	246	11.69	98	12.36	148	11.28	
Alcohol Intake, yes	273	12.98	93	11.74	180	13.73	0.21
Moderate drinking (1 to 3 days of drinking a week)	231	10.98	76	9.60	155	11.82	0.30
Heavy drinking (4 to 7 days of drinking a week)	42	2.00	17	2.15	25	1.91	
Refractive Error							
Myopia	522	24.80	186	23.46	336	25.61	0.90
Emmetropia	803	38.15	287	36.19	516	39.33	
Hyperopia	741	35.20	303	38.21	438	33.38	
Cataract Surgery	238	11.33	139	17.57	99	7.56	0.70
Axial length (mm)^¶^	22.76	3.99	22.44	4.47	22.95	3.66	0.02
Spherical equivalent^¶^	−0.07	2.06	0.11	1.76	−0.18	2.21	0.51
CFH rs10801555							
Non-carrier	700	33.25	300	37.83	400	30.49	0.25
Heterozygote	694	32.97	281	35.44	413	31.48	
Homozygote	176	8.36	65	8.20	111	8.46	
ARMS2 rs3750847							
Non-carrier	671	31.88	265	33.42	406	30.95	0.38
Heterozygote	717	34.06	294	37.07	423	32.24	
Homozygote	182	8.65	87	10.97	95	7.24	

SD = standard deviation;

^†^First generation participants are those who are born outside of Singapore (m = 793).

^††^Second generation participants are those who are born in Singapore, and whose parents are of Indian origins (n = 1312).

^*^Low economic status defined as participants with primary or lower education only and individual monthly income <$2,000 Singapore dollars. ^±^P-values is between first and second-generation immigrants. For continuous variables, adjusted t-tests for age were done; while for discrete variables, Logistic/Poisson Regression controlling for age were performed to check whether the two samples were having the same distribution.

^¶^Ocular values of affected eye. If not affected with AMD, then the right eye is taken. If both eyes are affected, then right eye is taken.

There are 793 first and 1312 second-generation immigrants who participated in SINDI-2. Compared to the first-generation immigrants, second-generation immigrants were younger, have a higher HbA1c, higher BMI, higher serum CRP, longer axial length and have higher social economic status (p < 0.05 for all).

### Incidence of AMD

The age-standardised 6-year incidence of early AMD was 5.26% (n = 107) and 0.51% (n = 10) for late AMD. 0.10% (n = 8) had geographic atrophy and 0.40% (n = 2) had neovascular AMD.

The age-standardized 6-year incidence of early and late AMD were not statistically different between first and second-generation immigrants (early AMD: 5.91% vs 4.21%, p = 0.37, and late AMD: 0.73% vs 0.25%, p = 0.11).

### Systemic Risk factors for incident AMD

In the multivariate model, age, cardiovascular disease history, underweight BMI, heavy alcohol intake and *ARMS2 rs3750847* homozygous genetic loci carrier remained significantly associated with the incident early AMD (P < 0.05 for all). Age and *ARMS2 rs3750847* homozygous genetic loci carrier remained significantly associated with incident early AMD in both first and second generations. Male gender was significantly associated with incident early AMD in first generation immigrants while underweight BMI was significantly associated with incident early AMD in second-generation immigrants (Table 2).

**Table 2:** Multivariable-adjusted Associations between Risk Factors and Incident Early AMD in SINDI II.

Variable*	Overall				First generation		Second generation	
	Model 1^†^ (N = 3994)		Multivariable adjusted^††^ (N = 3994)		Multivariable adjusted^†††^ (N = 1472)		Multivariable adjusted^†††^ (N = 2522)	
	Risk Ratio		Risk Ratio		Risk Ratio		Risk Ratio	
	(95% CI)	p-value	(95% CI)	p-value	(95% CI)	p-value	(95% CI)	p-value
Age, years	1.08 (1.06–1.1)	<0.001	1.07 (1.05–1.09)	<0.001	1.06 (1.03–1.09)	<0.001	1.08 (1.05–1.12)	<0.001
Sex								
Female	Ref	Ref	Ref	Ref	Ref	Ref	Ref	Ref
Male	0.87 (0.62–1.23)	0.43	0.67 (0.45–1.00)	0.05	0.55 (0.31–0.96)	0.04	0.84 (0.47–1.49)	0.55
Hypertension	1.34 (0.91–1.98)	0.14	1.26 (0.85–1.88)	0.25	1.11 (0.63–1.95)	0.72	1.35 (0.76–2.4)	0.31
Total cholesterol	0.88 (0.73–1.07)	0.20	0.98 (0.87–1.10)	0.70	0.91 (0.79–1.05)	0.18	1.08 (0.87–1.34)	0.49
Cardiovascular disease history	1.68 (1.11–2.54)	0.01	1.59 (1.04–2.45)	0.03	1.7 (0.96–3)	0.07	1.72 (0.88–3.34)	0.11
Body Mass Index (kg/m^2^), per SD	0.97 (0.8–1.18)	0.77	1.09 (0.87–1.38)	0.44	1.4 (0.86–2.28)	0.18	0.97 (0.69–1.35)	0.84
Underweight (16.0–18.5)	3.26 (1.43–7.41)	0.005	3.12 (1.37–7.14)	0.01	2.21 (0.72–6.76)	0.16	4.71 (1.29–17.26)	0.02
Normal (18.51–25)	Ref	Ref	Ref	Ref	Ref	Ref	Ref	Ref
Overweight (25.1–30)	1.04 (0.7–1.55)	0.84	1 (0.67–1.48)	0.99	0.74 (0.43–1.26)	0.27	1.52 (0.82–2.83)	0.18
Obese (>30)	0.99 (0.92–1.05)	0.67	1 (0.94–1.07)	0.97	1.03 (0.94–1.14)	0.49	0.95 (0.87–1.04)	0.29
Current smoker	1.13 (0.64–2.01)	0.67	0.87 (0.47–1.59)	0.64	1.2 (0.51–2.81)	0.68	0.74 (0.31–1.74)	0.49
Alcohol Frequency								
Non-drinker	Ref	Ref	Ref	Ref	Ref	Ref	Ref	Ref
Moderate drinker	1.71 (1.01–2.91)	0.05	1.68 (0.97–2.9)	0.06	1.23 (0.57–2.68)	0.60	2.15 (1–4.6)	0.05
Heavy drinker	3.05 (1.22–7.64)	0.02	3.14 (1.25–7.89)	0.02	2.63 (0.74–9.33)	0.13	3.39 (0.88–12.99)	0.08
Serum C-reactive Protein	1.01 (0.98–1.03)	0.68	1.01 (0.99–1.04)	0.33	0.99 (0.95–1.03)	0.63	1.03 (1–1.06)	0.09
Genetic Loci								
CFH								
Ref								
Heterozygote	1.15 (0.77–1.71)	0.50	1.21 (0.83–1.77)	0.32	1.5 (0.89–2.51)	0.13	1.01 (0.57–1.78)	0.97
Homozygote	0.8 (0.38–1.67)	0.55	0.9 (0.44–1.85)	0.77	1.45 (0.58–3.62)	0.43	0.52 (0.15–1.82)	0.31
ARMS2								
Ref								
Heterozygote	0.87 (0.56–1.35)	0.54	0.92 (0.62–1.38)	0.69	0.97 (0.55–1.72)	0.93	0.83 (0.47–1.49)	0.54
Homozygote	2.39 (1.45–3.95)	<0.001	2.52 (1.59–3.99)	<0.001	2.27 (1.22–4.21)	0.01	2.78 (1.36–5.68)	0.01

^**#**^Numbers are too small to generate any significant values in the analysis.

^†^Model 1: adjusting for age and gender.

^††^Multivariate model: adjusting for age, gender, hypertension, total cholesterol, cardiovascular disease, BMI categories, smoking status, alcohol consumption frequency and ARMS2 genetic loci.

^†††^Multivariate model: adjusting for age, gender, hypertension, total cholesterol, cardiovascular disease, BMI categories, smoking status, alcohol consumption frequency, serum C-reactive protein and ARMS2 genetic loci.

### Ocular Risk factors for incident AMD

The age-standardized six-year incidence of neovascular AMD was highest for participants with baseline soft indistinct drusen and pigment (4.72%), followed by those with baseline pigmentary abnormalities (0.87%), intermediate drusen without pigmentary abnormalities (0.33%) and was lowest in those with no AMD at baseline (0.16%) (P < 0.05) (Data not shown). We were not able to assess associations between any of the baseline ocular features with incident GA, due to a very small number of events (n = 2).

## Discussion

The six-year age-standardised incidence of early and late AMD was 5.26% and 0.51% respectively in this Singaporean Indian population-based sample. Both first and second-generation immigrants have similar incidence rates of early and late AMD.

The six-year age-standardized cumulative incidence in this Indian population is lower than that of Caucasian populations which range from 8.19%^7^ to 8.74%^8^ for early AMD and 0.19%^7^ to 1.10%^8^ for late AMD. Rates are comparable to earlier Asian populations; the Singapore Malay Eye Study^15^ (SiMES) reported 6-year incidence of 6.13% and 0.83% for early and late AMD respectively. The Beijing Eye Study^13^ reported 5-year early and late AMD incidence at 2.60% and 0.10% respectively. The Hisayama study^14^ reported 5-year early and late AMD incidence at 8.5% and 0.87% respectively. The SiMES and Beijing studies however had similar mean age at baseline of its study population at 54 years old, compared to our study’s, which is lower than that of the Hisayama’s study at 60 years old. Age range remains an important consideration when comparing the incidence of AMD across studies.

Older age, underweight BMI, cardiovascular disease history, higher alcohol intake and *ARMS2 rs3750847 homozygous* genetic loci were associated with an increased risk of incident early AMD.

Our finding of an association between underweight BMI and early incident AMD is similar to previous studies. In the Blue Mountains Eye Study^8^, having a BMI of either lower or higher than the accepted normal range of 20–25 kg/m^2^ was associated with a significantly increased risk of early AMD. In the Physician’s Health Study^50^, it reported a J-shaped association between BMI and the incidence of visually significant AMD with the highest incidence among obese men with a BMI > 30 and a lower incidence among the leanest men with BMI < 22. Perhaps deficiencies in important macronutrients such as carotenoids in the diets of underweight people could lead to a higher risk of AMD^51^. However, this speculation would warrant future studies. Evidence for a history of cardiovascular disease and its association with AMD remains inconsistent^52–55^, with chronic inflammation being a possible shared biomechanism for both AMD and cardiovascular disease^56^.

Heavy alcohol consumption (more than three standard drinks a day) is known to be associated with an increased risk of early AMD of In the Western population^57^. Alcohol is a known neurotoxin that can result in oxidative brain damage^58,59^, and could compromise mechanisms which protect against oxidative stress in the retina leading to AMD^60,61^. We could not evaluate the dose-response curve between alcohol consumption and AMD as we lacked data on alcohol units and types of alcoholic beverage consumed a day.

Homozygous carrier of the *ARMS2 rs3750847* genetic loci was significantly associated with incident early AMD. This is similar to the finding from the GAMA consortium which showed associations of *ARMS2 rs3750847* with Asian AMD in a genome-wide association study^43^ and previous Asian studies^62–64^. The role of *CFH* in early AMD from previous Asian genetic studies remains controversial^65–67^. Our finding adds to existing work and suggest *ARMS2* may have a stronger influence on AMD risk in Asians than *CFH*, owing to the low frequency of the risk allele Y402H (rs1061170) variant in Asians^68–71^. Extensive epidemiologic and genetic analyses have led to the conclusion that AMD results from the complex interplay of multiple environmental and genetic factors which in combination account for the development of the phenotype. The high prevalence of the disease implies that interactions amongst multiple genetic and environmental factors influence an individual’s susceptibility to AMD. In our study, there were significant differences in baseline characteristics between the first-generation and second-generation immigrants (HbA1c, obesity, serum CRP and socioeconomic status). Despite the obvious differences in systemic risk profiles, the incidences of early and late AMD and patterns of risk factors for early AMD were interestingly similar between the two generations. This observation is in keeping with our previous publication which reported similar prevalence of AMD among Indian adults living in urban Singapore and rural India despite diverse differences in environmental and systemic risk factor profiles^47^. Perhaps genetic inheritance, compared to environmental and systemic risk factors, has greater contribution to the risk of developing AMD in Indians. Chakravarthy *et al*. identified in a meta-analysis that a family history of AMD showed a stronger association with late AMD in comparison to other environmental risk factors such as smoking and a higher BMI^72^. A previous study in Koreans showed that the presence of ARMS2, but not CFH rs800292 genetic loci, is associated with a greater risk of having exudative AMD compared to other risk factors of spherical equivalent and smoking^73^. Despite recent studies in AMD genetics which established alleles and haplotypes on chromosome 1 in CFH and on chromosome 10 in ARMS2 as having large influences on the risk for all AMD subtypes in populations of various ethnicities, the combination of these genes alone has been shown to be insufficient to correctly predict the development and progression of this disease^74,75^. It remains a challenge to assess the effect size of each specific genetic and environmental factors on the risk of AMD. Certain factors may affect predominantly the incidence of late AMD, which would require a much larger sample size to evaluate. The importance of genetic awareness though allows for Individuals with known high-risk genes to be counselled with regards to smoking cessation and also to have a heightened level of self-monitoring of vision. Eye screening in individuals with a strong family history of AMD would also be critical for earlier detection and treatment of disease.

Strengths of the current study include a large population-based, multi-ethnic sample with standardized grading of fundus images and methodology similar to that used in other earlier landmark studies such as the Beaver Dam Eye Study and the Blue Mountain Eye Study for comparison of findings. However, there were limitations to the study as well. Participants lost to follow-up had higher blood pressure, poorer sugar control and were more likely to be living alone. These could be significant risk factors for AMD in the longer run. Also, we only managed to identify 2 participants at the follow-up study with reticular drusen in our entire cohort. This is likely an under-estimation of reticular drusen on colour fundus photo, and hence we decided not to include ‘reticular drusen’ in our eventual analysis. Ideally, we would have collected the blood samples in the fasting state, however logistically it is not feasible in a population study. Nonfasting lipid is believed to be also informative as recent studies, as well as the European Atherosclerosis Society and European Federation of Clinical Chemistry and Laboratory Medicine have stated that fasting is not essential for determining lipid profile in relation to predicting cardiovascular disease. most humans spend their day in the nonfasting state, and the nonfasting state might actually be more physiologically relevant in health and disease that the fasting state^76–78^.

In summary, the incidence of early and late AMD in this Singaporean Indian population is lower than that in Caucasian populations. Systemic risk factors for six-year incident early AMD include underweight BMI, cardiovascular disease history, heavy alcohol intake and *ARMS2 rs3750847* homozygous genetic loci. Presence of drusen and pigmentary changes at baseline are associated with incidence of late AMD. Although second-generation immigrants have an increased incidence of systemic vascular disease at baseline compared to the first-generation immigrants, both generations appear to have similar incidence of early and late AMD and risk factor patterns of early AMD, suggesting that genetic inheritance, compared to environmental and systemic risk factors, has greater contribution to the risk of developing early AMD in Indians.

## Electronic supplementary material

Comparison of Baseline Characteristics between Participants observed and not observed at 6-year examination in the Singapore Indian Study (SINDI)
