# Supplementary material for: Six-Year Incidence and Risk Factors of Age-Related Macular Degeneration in Singaporean Indians: The Singapore Indian Eye Study
Source: Sci Rep. 2018 Jun 11;8:8869. doi: 10.1038/s41598-018-27202-w (PMC5995892; doi:10.1038/s41598-018-27202-w)
Supplement: Supplementary file 1 — Comparison of Baseline Characteristics between Participants observed and not observed at 6-year examination in the Singapore Indian Study (SINDI) [file 41598_2018_27202_MOESM1_ESM.docx]

**Six-Year Incidence and Risk Factors of Age-Related Macular Degeneration in Singaporean Indians: The Singapore Indian Eye Study**

Valencia Hui Xian Foo^1^, Yasuo Yanagi^1,2^, Nguyen Duc Quang^1^,

Charumathi Sabanayagam^1,3^, Lim Sing Hui^4^, Kumari Neelam^1,2^,

Jie Jin Wang^5^, Paul Mitchell^6^, Ching-Yu Cheng^1,2,3^

Tien Yin Wong^1, 2, 4^, Chui Ming Gemmy Cheung^1, 2, 4^

1. Singapore Eye Research Institute, Singapore National Eye Centre, Singapore
2. Ophthalmology & Visual Sciences Academic Clinical Program (Eye ACP), Duke-NUS Medical School, Singapore
3. Centre for Quantitative Medicine, Duke-NUS Medical School, Singapore
4. Singapore National Eye Centre, Singapore
5. Academic Medicine Research Institute, Duke-NUS Medical School, Singapore, Singapore.
6. Centre for Vision Research, Westmead Millennium Institute of Medical Research, University of Sydney, Westmead, Sydney, Australia.

**Correspondence to:**

A/Prof Gemmy Cheung

Singapore Eye Research Institute, Singapore National Eye Centre

11 Third Hospital Avenue, Singapore 168751

Phone: (65) 69881460, Fax: (65) 62263995

Email Address: gemmy.cheung.c.m@singhealth.com.sg

**Funding/Support (including none):** National Medical Research Council grants no. 0796/2003 and Biomedical Research Council Grant no. 501/25-5.

**Financial Disclosures**: No conflicting relationship exists for any author.

**Running Head:** Six year incidence and risk factors of AMD in Singaporean Indians

**Keywords**: population cohort, age-related macular degeneration, risk factors, drusen, pigmentary abnormalities

Abstract word count: 200 words

Manuscript word count: 2943 words

**Supplementary Table 1. Comparison of Baseline Characteristics between Participants observed and not observed at 6-year examination in the Singapore Indian Study (SINDI)**

| Variable* | Participants at both examinations | | Not observed or no retinal photographs ^†^ | | P Value^±^ |
| --- | --- | --- | --- | --- | --- |
|  | (n=2105) | | (n= 809) | |  |
|  | Mean or n (%) | SD or % | Mean or n (%) | SD or % |  |
| Age (years) | 56.20 | 9.07 | 56.76 | 9.66 | 0.14 |
| Sex, Male | 1047 | 49.74 | 392 | 48.45 | 0.65 |
| Hypertension, yes | 1177 | 55.99 | 498 | 61.71 | 0.17 |
| Systolic Blood Pressure, (mmHg) | 134.11 | 19.30 | 136.88 | 20.86 | <0.001 |
| Diastolic Blood Pressure, (mmHg) | 77.75 | 10.11 | 78.43 | 10.60 | 0.08 |
| Diabetes | 570 | 27.17 | 233 | 29.02 | 0.63 |
| Hba1c, % | 6.36 | 1.32 | 6.49 | 1.51 | 0.03 |
| Chronic Kidney Disease | 105 | 5.16 | 50 | 6.45 | 0.41 |
| Blood Creatinine (mmol/l) | 0.85 | 0.25 | 0.86 | 0.39 | 0.95 |
| History of Myocardial Infarction | 162 | 7.70 | 66 | 8.19 | 0.97 |
| History of Stroke | 43 | 2.04 | 19 | 2.36 | 0.68 |
| Body Mass Index (kg/m^2^) | 26.20 | 4.48 | 26.32 | 4.91 | 0.48 |
| Underweight | 28 | 1.33 | 15 | 1.85 | 0.64 |
| Normal | 877 | 41.66 | 328 | 40.54 |  |
| Overweight | 849 | 40.33 | 307 | 37.95 |  |
| Obese | 347 | 16.48 | 157 | 19.41 |  |
| Total Cholesterol (mg/dl) | 5.21 | 1.07 | 5.28 | 1.14 | 0.06 |
| HDL Cholesterol (mg/dl) | 1.06 | 0.31 | 1.07 | 0.32 | 0.47 |
| LDL Cholesterol (mg/dl) | 3.35 | 0.93 | 3.39 | 0.96 | 0.20 |
| Triglycerides (mg/dl) | 1.96 | 1.17 | 2.01 | 1.19 | 0.29 |
| C-reactive Protein (mg/l), mgL | 4.08 | 5.75 | 4.42 | 6.53 | 0.18 |
| Formal Education, yes | 1787 | 84.97 | 665 | 82.40 | 0.74 |
| Low income, <$2000 | 1440 | 70.24 | 586 | 74.74 | 0.49 |
| Low Economic Status*, yes | 967 | 47.19 | 418 | 53.32 | 0.07 |
| Living alone | 87 | 4.14 | 51 | 6.32 | 0.02 |
| Cigarette Smoking status |  |  |  |  | 0.81 |
| Never smoked | 1570 | 74.58 | 591 | 73.05 |  |
| Current smoker | 285 | 13.54 | 142 | 17.55 |  |
| Past Smoker | 246 | 11.69 | 74 | 9.15 |  |
| Alcohol Intake | 273 | 12.98 | 110 | 13.65 | 0.65 |
| Alcohol Intake frequencies |  |  |  |  | 0.35 |
| No alcohol intake | 1830 | 87.02 | 696 | 86.35 | 0.35 |
| Moderate drinkers | 231 | 10.98 | 87 | 10.79 |  |
| Heavy drinkers | 42 | 2.00 | 23 | 2.85 |  |
| Refractive Error |  |  |  |  | 0.92 |
| Myopia | 522 | 24.80 | 192 | 23.73 |  |
| Emmetropia | 803 | 38.15 | 299 | 36.96 |  |
| Hyperopia | 741 | 35.20 | 284 | 35.11 |  |
| Cataract Surgery | 238 | 11.33 | 82 | 10.19 | 0.09 |
| Axial length (mm) ¶ | 22.76 | 3.99 | 22.81 | 3.81 | 0.70 |
| Spherical equivalent¶ | -0.07 | 2.06 | -0.17 | 2.41 | 0.19 |
| Early AMD at baseline | 100 | 4.75 | 43 | 5.80 | 0.32 |
| Late AMD at baseline | 8 | 0.38 | 2 | 0.27 | 0.64 |
| Immigration status |  |  |  |  |  |
| 1^st^ generation | 793 | 37.67 | 35 | 36.84 | 0.14 |
| 2^nd^ generation | 1312 | 62.33 | 60 | 63.16 | 0.20 |
| CFH |  |  |  |  |  |
| Non-carrier | 700 | 33.25 | 287 | 35.48 | 0.54 |
| Heterozygote | 694 | 32.97 | 245 | 30.28 |  |
| Homozygote | 176 | 8.36 | 71 | 8.78 |  |
| ARMS2 |  |  |  |  |  |
| Non-carrier | 671 | 31.88 | 251 | 31.03 | 0.50 |
| Heterozygote | 717 | 34.06 | 271 | 33.50 |  |
| Homozygote | 182 | 8.65 | 81 | 10.01 |  |

SD=standard deviation;

† Includes subjects (n= 809) who participated in SINDI I but did not have retinal photography data / ungradable images collected to be included in SINDI II (n=323); and subjects who are ineligible (ineligible: uncontactable, deceased, terminally ill, have psychiatric illnesses, in prison or has migrated) due to lost to follow-up after SINDI I (n= 486).

Bolded values indicate a statistically significant result (p<0.05) for tests of differences between the group with AMD data at SINDI II and the specific group without AMD data at SINDI II or ineligible due to lost to follow-up to be included in SINDI II (n=486).

*Low economic status defined as participants with primary or lower education only and individual monthly income <$2,000 Singapore dollars

± P-values is between baseline of the first (“participants at both examinations”) and second groups (“Not observed or no retinal photographs”) of patients. For Continous variables, t-tests were done; while for discrete variables, Mann–Whitney U tests were performed to check whether the two samples were having the same distribution.

¶ Taken from the affected AMD eye. If not affected with AMD, then the right eye is taken. If both eyes are affected, then right eye is taken
